# Excessive boron fertilization-induced toxicity is related to boron transport in field-grown pomelo trees

**DOI:** 10.3389/fpls.2024.1438664

**Published:** 2024-09-10

**Authors:** Ziwei Luo, Lijun Zhang, Wenlang Hu, Yuwen Wang, Jingxia Tao, Yamin Jia, Ruizhen Miao, Li-Song Chen, Jiuxin Guo

**Affiliations:** ^1^ Fujian Provincial Key Laboratory of Soil Environmental Health and Regulation, International Magnesium Institute, College of Resources and Environment, Fujian Agriculture and Forestry University, Fuzhou, China; ^2^ College of Agriculture, Guangxi University, Nanning, China; ^3^ Forestry Science and Technology Test Center of Fujian Province, Zhangzhou, China; ^4^ College of Forestry, Guangxi University, Nanning, China

**Keywords:** B toxicity, citrus plants, mineral profiles, fruit yield and quality, B biochemical cycle

## Abstract

Boron (B) is an essential micronutrient for plant growth and development; however, the process of B toxicity in citrus production is still poorly understood. We proposed a hypothesis that B toxicity in citrus trees is related to the characteristics of B transport from soil to leaf or fruit. For this, a field experiment was conducted for two treatments, control (B free or without B) and B fertilizer treatment (100 g Na_2_B_4_O_7_·10H_2_O plant^−1^), to investigate the effects on plant growth, nutrient uptake, fruit yield and quality, and B transport in 10-year-old pomelo trees [*Citrus grandis* (L.) Osbeck cv. Guanximiyou]. Our results showed that excess B fertilization directly led to B toxicity in pomelo trees by dramatically increasing soil total B and water-soluble B contents. B toxicity induced interveinal chlorosis in leaves and decreased leaf biomass and function, resulting in a decreased 45.3% fruit yield by reducing 30.6% fruit load and 21.4% single fruit weight. Also, B toxicity induced changes in mineral elements between leaf positions and fruit parts, in which the concentrations of B, potassium, and magnesium were increased while those of nitrogen and iron were decreased. Under B toxicity conditions, fruit quality parameters of total soluble solids (TSS), TSS/titratable acidity (TA), total soluble sugar, sucrose, pH, vitamin C, and total phenol contents decreased, which were regulated by the lower carbohydrate production in new leaves and the lower transport capacity in old leaves. Moreover, B toxicity significantly increased the transfer factor and bio-concentration factor of B in pomelo plants, with higher levels in leaf organs than in fruit organs. Taken together, excess B fertilization-induced B toxicity in pomelo trees, with induced growth inhibition and nutrient disorder, results in reduced fruit yield and quality, which are related to B transport from soil to organs. The findings of this study highlight the understanding of B toxicity in citrus plants and strengthen B management in pomelo production for high yield and high quality.

## Highlights

- Excess boron (B) fertilization induced B toxicity in field-grown pomelo trees.
- B toxicity induced growth inhibition and nutrient disorder by increasing B accumulation.
- B toxicity reduced fruit yield and quality by mediated source–sink balance.
- The effects of B toxicity on pomelo trees are related to B transport from soil to organs.

## 1 Introduction

Boron (B) is an indispensable microelement for plant growth and development, which plays important roles in diverse physiological and biochemical functions, including cell elongation, cell wall formation, carbohydrate and energy metabolism, cell membrane integrity, gene expression, enzyme activity, phenol accumulation, pollen tube growth, and crop yield and quality formation (Shah et al., 2017; Brdar-Jokanović, 2020; García-Sánchez et al., 2020; Li et al., 2023). Low B availability in soils directly inhibits vegetative and reproductive growth, and the adverse effects of B deficiency in plants are widely important (Brdar-Jokanović, 2020; Yang et al., 2022). Although sufficient B supply is the basis for crop yield formation and quality improvement during a whole growth period, the threshold of B nutrition is narrow between deficiency and toxicity (Shah et al., 2017; Brdar-Jokanović, 2020; García-Sánchez et al., 2020). Favorably, the theoretical and applied research on B deficiency and its correction in the soil–plant system has received increasing attention from researchers and growers (Mattos-Jr et al., 2017; Shah et al., 2017; Riaz et al., 2018a; Guo et al., 2022; Zhou et al., 2022). Unfortunately, B toxicity often occurs in agricultural production due to unreasonable practices, such as excess B-contained fertilization, B-rich water irrigation, and B-contaminated soil in the tailing pond, directly affecting the yield and quality of many crops, including citrus (Landi et al., 2019; Yang et al., 2022).

Citrus is one of the most important fruit crops, with rich germplasm resources, such as oranges, grapefruits, mandarins, tangerines, lemons, and pomelos (Liu Y. Q. et al., 2012). Due to the expanding planting practices for high economic return and growing consumer demand for high nutrition value, citrus is gradually developing into the world’s largest fruit crop. In 2022, the citrus planting area and fruit yield were approximately 7.2 × 10^6^ ha and 1.2 × 10^8^ t in the world, respectively, reaching 8.0 × 10^5^ ha and 2.2 × 10^7^ t in China (FAOSTAT, 2023). The lack of scientific knowledge about B fertilization matches plant demand with B stress frequently observed in citrus production, resulting in the loss of productivity and fruit quality (Cheng et al., 2022; Yang et al., 2022). B toxicity originating from unreasonable measures of B deficiency correction in citrus production has attracted increasing attention. Guo et al. (2014) revealed that *Citrus sinensis* was more tolerant to B toxicity than *C. grandis* at the gene expression level, and their miRNA characteristics were also identified by Huang et al. (2019). Considering the differences in B tolerance, the response mechanism and potential value of different citrus cultivars or rootstocks to B stress have been gradually revealed (Simón-Grao et al., 2018). Papadakis et al. (2004a) reported that the *Citrus clementina* grafted on Swingle citrumelo was more tolerant to B toxicity than sour orange according to various morphological and physiological characteristics. These results demonstrated that citrus plants are sensitive to B excess, in which toxicity causes major physiological and metabolic disorders.

Huang et al. (2014) reported that B toxicity induced programmed cell death of leaf phloem tissue, and higher free B concentration may contribute to the lower B tolerance between citrus species. Wu et al. (2018) reported that B toxicity caused alterations in the central metabolism pattern in two citrus seedlings between the upper and lower leaves due to the symptoms of B toxicity being preferentially manifested in the lower leaves. Regardless of cultured conditions, the accumulation degree of B concentration in citrus plants has yet to be studied adequately under B toxicity conditions, especially in different leaf positions and ages. Also, differences in citrus seedlings between leaf tip and leaf center were demonstrated, including pectin network crosslinking structure, cell wall integrity, protein structure, carbohydrate, and cellulose content (Wu et al., 2019). Despite the importance of B nutrition for crop productivity, the mechanisms by which citrus plants’ fruit yield and quality formation respond to B toxicity need to be better understood, especially in B concentration in different fruit parts for dietary health (Brdar-Jokanović, 2020; García-Sánchez et al., 2020; Hua et al., 2021; Li et al., 2023). Therefore, it is necessary to reveal the negative effect of B toxicity on citrus plants in field conditions, which helps to improve B management strategies in citrus production (Yang et al., 2022).

Based on the above concerns, the objectives of this study were to investigate the effects of B toxicity on growth variations and nutrient profiles in different leaf positions and fruit parts, to reveal the impact of B toxicity on fruit yield and quality, and to address the characteristics of B transport from soil to plant in a pomelo field condition. Also, integrated B management for sustainable citrus production was discussed.

## 2 Materials and methods

### 2.1 Experimental design

The field experiment was conducted in Pinghe County (24.36°N, 117.31°E), Fujian Province, southern China, the highest concentrated pomelo production region. As one of the most famous citrus varieties, ‘Guanximiyou’ pomelo [*C. grandis* (L.) Osbeck] has high nutritional and medicinal value, especially red-fleshed pomelo (Wang et al., 2014, 2019). In this study, red-fleshed pomelo trees grafted onto sour pomelo trees were planted in 2009, with the soil type geared to the red soil, and the parent material is ferralsol (Food and Agriculture Organization, FAO). According to the method described by Guo et al. (2022), the 0–40-cm soil physical and chemical properties were pH 4.23, 6.39 g kg^-1^ organic matter, 1.12 g kg^-1^ total nitrogen (N), and available nutrients, including 444.00 mg kg^-1^ phosphorus (P), 326.22 mg kg^-1^ potassium (K), 159.26 mg kg^-1^ calcium (Ca), and 55.06 mg kg^-1^ magnesium (Mg), and 0.51 mg kg^-1^ B. Based on the previous reports (Guo et al., 2019, 2022; Thakur et al., 2023), the soil available B content in the study belongs to the critical B level. The planted density of pomelo trees was approximately 750 plants ha^−1^, and the trees were spaced approximately 3.5–4.0 m × 3.5–4.0 m spacing.

In 2018, a field experiment was conducted for two treatments: control (B free or without B) and B fertilizer treatment (100 g Na_2_B_4_O_7_·10H_2_O plant^−1^). Six representative pomelo trees per treatment were selected, with three blocks and two trees in each replicate. The fertilization scheme was adopted for high-yield and high-quality pomelo production, with a total of 450 kg N ha^−1^, 450 kg P_2_O_5_ ha^−1^, and 450 kg K_2_O ha^−1^ split into four equal rates and applied at the basal (December 2018), shooting and flowering (February 2019), fruit setting (April 2019), and fruit expanding stages (June 2019). In the B fertilizer treatment, 100 g borax plant^−1^ (as Na_2_B_4_O_7_·10H_2_O) was mixed in the NPK compost fertilizer (as N–P_2_O_5_–K_2_O was 15–15–15) and divided equally into two parts, which were added in the shooting and flowering stages. According to the method described by Zhang et al. (2021), all fertilizers were circularly broadcast and applied at the drip line of the tree crown (1.0–1.5 m from the stem base). The practice management, including diseases and insect pests, was performed in experimental progress in the pomelo orchard, and no other micronutrient fertilizers were used.

### 2.2 Soil B concentration measurement

At the harvest stage (October 2019), the soil samples were collected from the soil tillage depths of 0–20 cm and 20–40 cm. Each soil sample was a composite of four sub-samples taken in a pomelo tree across treatments. The soil samples were air-dried, ground, and sieved for soil B measurement (Guo et al., 2019, 2022). The soil B concentrations were divided into water-soluble B and total B, extracted by hot water and sodium carbonate, respectively; then, B concentrations in the extracted solutions were determined using the curcumin colorimetric method. Each treatment was repeated six times. A red soil sample (GBW(E)070045) was a certified reference material from the Institute of Geophysical and Geochemical Exploration, Chinese Academy of Geosciences, with the standard value of total B content of 71 ± 9 mg kg^-1^.

### 2.3 Leaf growth characteristics

At the harvest stage, the leaf samples were divided into four types in different leaf positions, including new leaves with fruit (NL+F), old leaves with fruit (OL+F), new leaves without fruit (NL−F), and old leaves without fruit (OL−F), in which the new and old leaves represent annual and biennial leaves, respectively. Leaf samples in 1/2 shoot length were selected, with two leaves per shoot and four shoots per tree, resulting in eight leaves per leaf position collected from the mid-upper canopy around the tree in the center of an orchard. Each treatment or leaf type was repeated six times, with one replicate containing eight leaves.

For leaf growth characteristics, the relative chlorophyll content [soil plant analysis development (SPAD) value] was determined using a SPAD-502 portable meter (Minolta Camera, Osaka, Japan), which measured two points along the middle of both sides of the main leaf vein, with the mean value of four data per leaf recorded (Chen et al., 2020). The leaf water content was calculated by fresh weight and dry weight. The leaf area was measured in combination with a DR-6030C Scanner (Canon, Beijing, China) and Image-Pro Plus 6.0 (Media Cybernetics, Silver Spring, MD, USA), and the specific leaf weight (SLW; leaf dry weight/leaf area) was further analyzed.

### 2.4 Leaf carbohydrate measurement

The total soluble sugar and starch contents in the leaf were determined using 630 g L^−1^ ethanol and 9.2 mol L^−1^ HClO_4_ extracted, respectively, and measured using an anthrone-ethyl acetate colorimetry method (Ribeiro et al., 2012). Each treatment or leaf type was repeated six times. As a result, the non-structural carbohydrate (NSC) content was a sum of total soluble sugar and starch.

### 2.5 Fruit growth characteristics

Similar to leaf samples, two fruit samples per tree were collected in the middle of the outer canopy from two directions, east and west, as fruit samples and divided into pulp and peel parts for further analysis (Zhang et al., 2021; Yan X. et al., 2024). Fruit growth characteristics, including fruit load (or the number of fruits per tree), single fruit fresh weight, vertical and horizontal diameters, and pulp and peel dry weight, were measured. Additionally, the fruit shape index (vertical diameter/horizontal diameter), edible rate (pulp fresh weight/fruit fresh weight), pule and peel water content, and the dry weight proportion of pulp and peel were further calculated. Each treatment was repeated six times, with one replicate containing two individual fruits.

### 2.6 Fruit quality parameters

According to the standard methods (Gullo et al., 2020), fresh pulp samples were adopted to determine some physiology and biochemistry parameters for fruit quality, including total soluble solids (TSS), titratable acidity (TA), TSS/TA, total soluble sugar, sucrose, sucrose percent (sucrose/total soluble sugar × 100), pH, vitamin C (Vc), and total phenol content. Each treatment was repeated six times.

### 2.7 Leaf and fruit mineral analysis

To determine the mineral concentrations, including N, P, K, Ca, Mg, Fe, Mn, and Cu, the dried and ground samples of leaf and fruit were completely digested by the HNO_3_–HClO_4_ method (4:2 v), except for N analysis by the H_2_SO_4_–H_2_O_2_ method (Chen et al., 2020). N concentration was measured by AA3 digital colorimeter (Bran + Luebbe, Hamburg, Germany), and other mineral elements were determined using Optima 7300 DV inductively coupled plasma–optical emission spectrometer (ICP-OES; PerkinElmer, Waltham, MA, USA). Also, according to the method described by Wu et al. (2019), B concentration was measured by the curcumin colorimetric method at a 540-nm spectrophotometer (Libra S22, Biochrom, Cambridge, UK). Each treatment was repeated six times. A citrus leaf sample (GBW10020) was a certified reference material from the Institute of Geophysical and Geochemical Exploration, Chinese Academy of Geosciences, with the standard value of total B content of 32 ± 3 mg kg^-1^.

### 2.8 Transfer factor and bio-concentration factor of B

The transfer factor (TF) and bio-concentration factor (BCF) are the ratios of the concentration of a mineral element in two parts of the plant–soil system (Zhang et al., 2018). The values of TFs and BCFs of B from soil to organ or from organ to organ in pomelo plants between control and B toxicity treatments were calculated using the following equations:


(1) $$TF=C_{organ}/C_{soil\;or\;another\;organ}$$

(2) $$BCF=\left(C_{B\;toxicity\;organ}–C_{control\;organ}\right)/\left(C_{B\;toxicity\;soil}–C_{control\;soil}\right)$$

where C_organ_ and C_soil or another organ_ refer to the B concentrations in organ and soil samples, respectively; the C_B toxicity organ_ and C_control organ_ refer to the B concentrations in a specific organ of the pomelo between B toxicity and control treatments, respectively; C_B toxicity soil_ and C_control soil_ represent the B concentrations in the B toxicity soil and control soil, respectively. Also, soil B concentration in these equations was estimated using the soil water-solution B and total B concentration, respectively. Each treatment was repeated six times.

### 2.9 Statistical analysis

Data on pomelo growth and quality parameters were subjected to single-factor analysis of variance (ANOVA) to evaluate the effects of B toxicity. The differences in mean comparisons were performed between the control and B fertilizer treatment using the least significant difference (LSD) test, with *p*< 0.05 considered to indicate statistical significance by one-way ANOVA. All statistical analyses were performed using the SAS 9.3 software (SAS Institute, Cary, NC, USA). The principal component analysis (PCA) was conducted using the Origin Pro 2022b (OriginLab Corp., Northampton, MA, USA). The structural equation modeling (SEM) analysis was performed using the SmartPLS 4 (SmartPLS GmbH, Oststeinbek, Germany), and the data were fitted into the model using the partial least squares method.

## 3 Results

### 3.1 B toxicity reduces leaf and fruit growth characteristics

**Figure 1:**
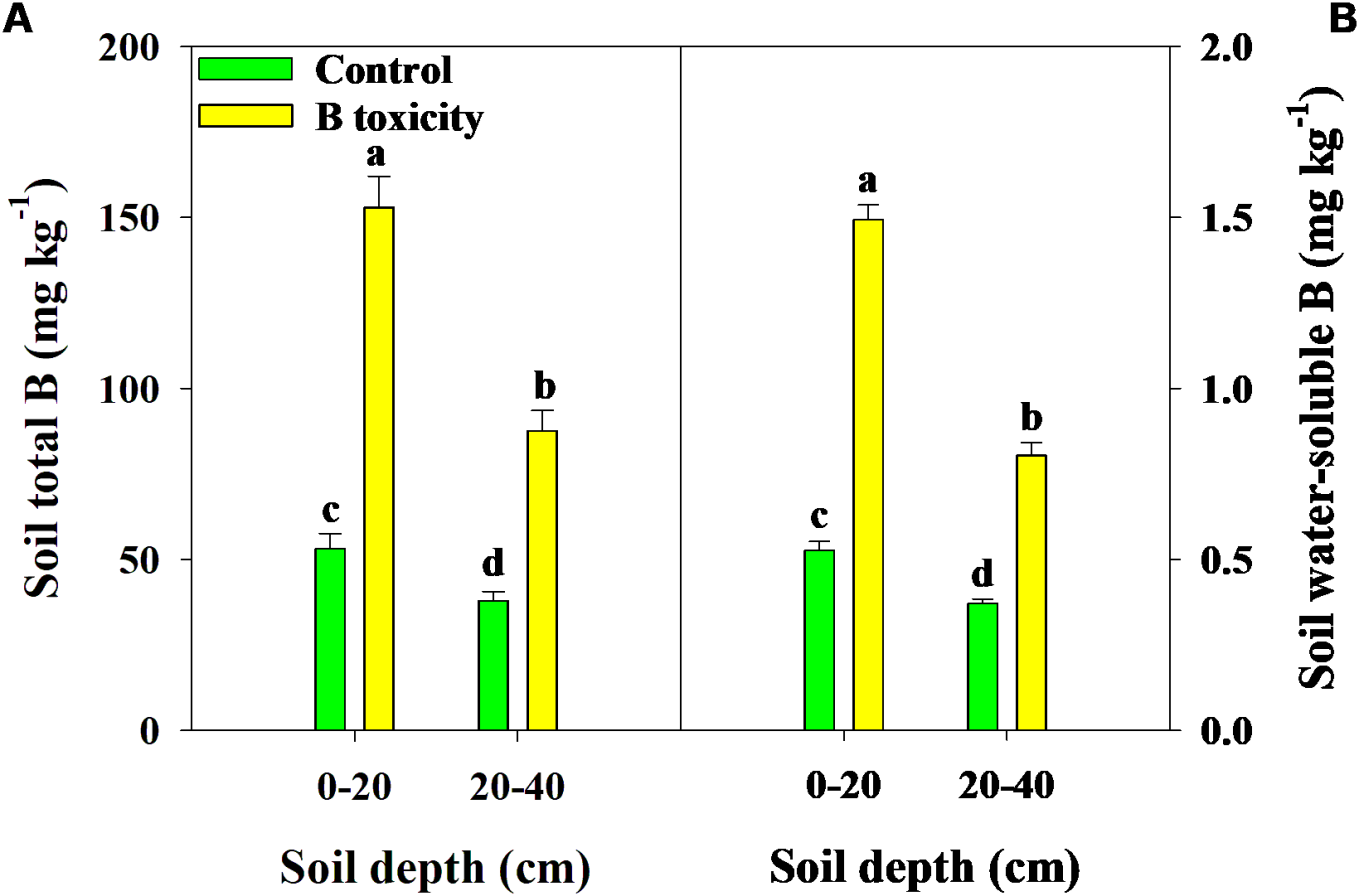
Effects of excess B fertilization on concentrations of soil total B **(A)** and soil water-soluble B **(B)** in soil depths of 0–20 and 20–40 cm. The error bars indicate standard deviation, statistical analysis was carried out by ANOVA plus least significant difference (LSD) test, and statistical significance (*p*< 0.05) is indicated with different lowercase letters (a–d) in the different soil depths.

**Figure 2:**
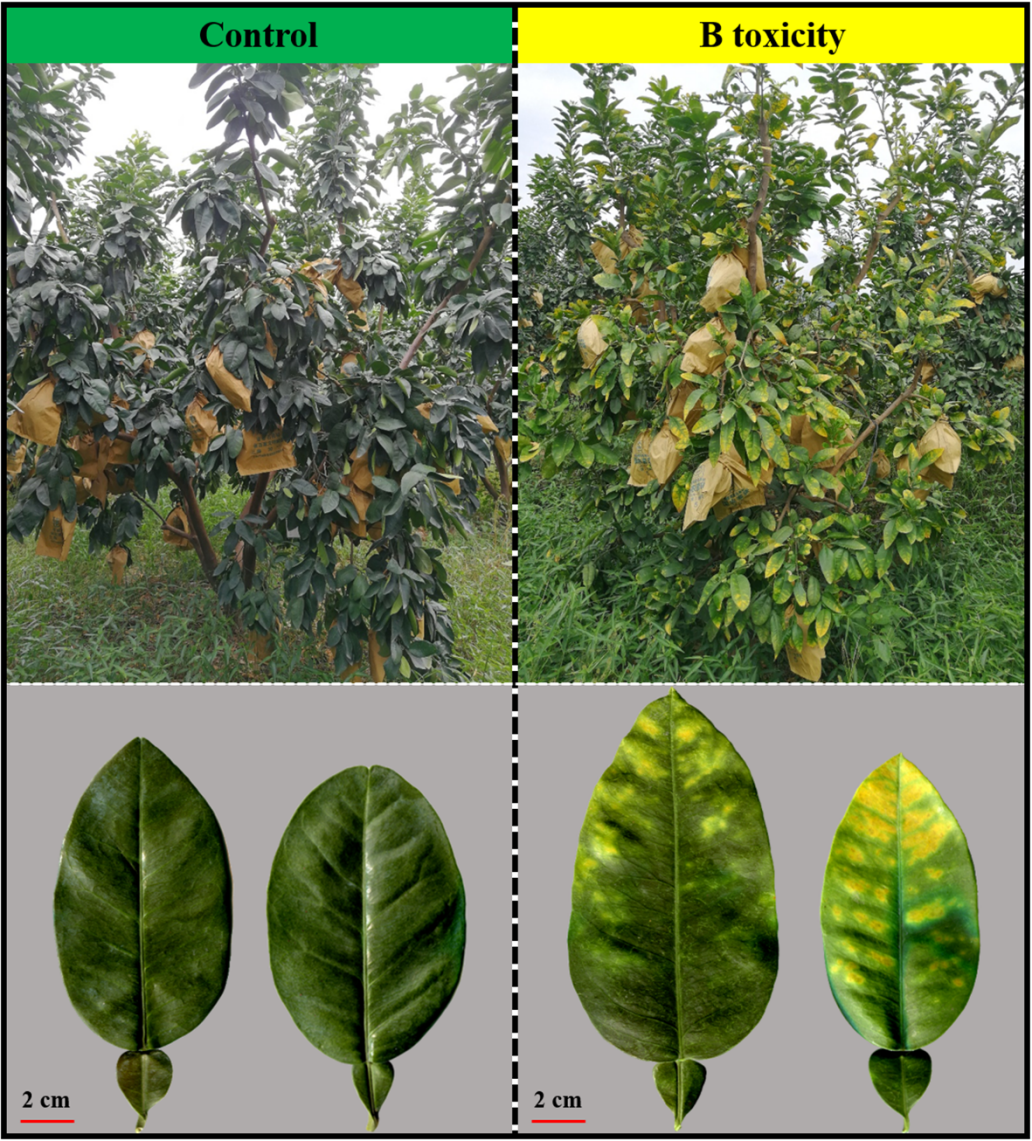
Field symptoms of the control and B toxicity in pomelo trees.

**Figure 3:**
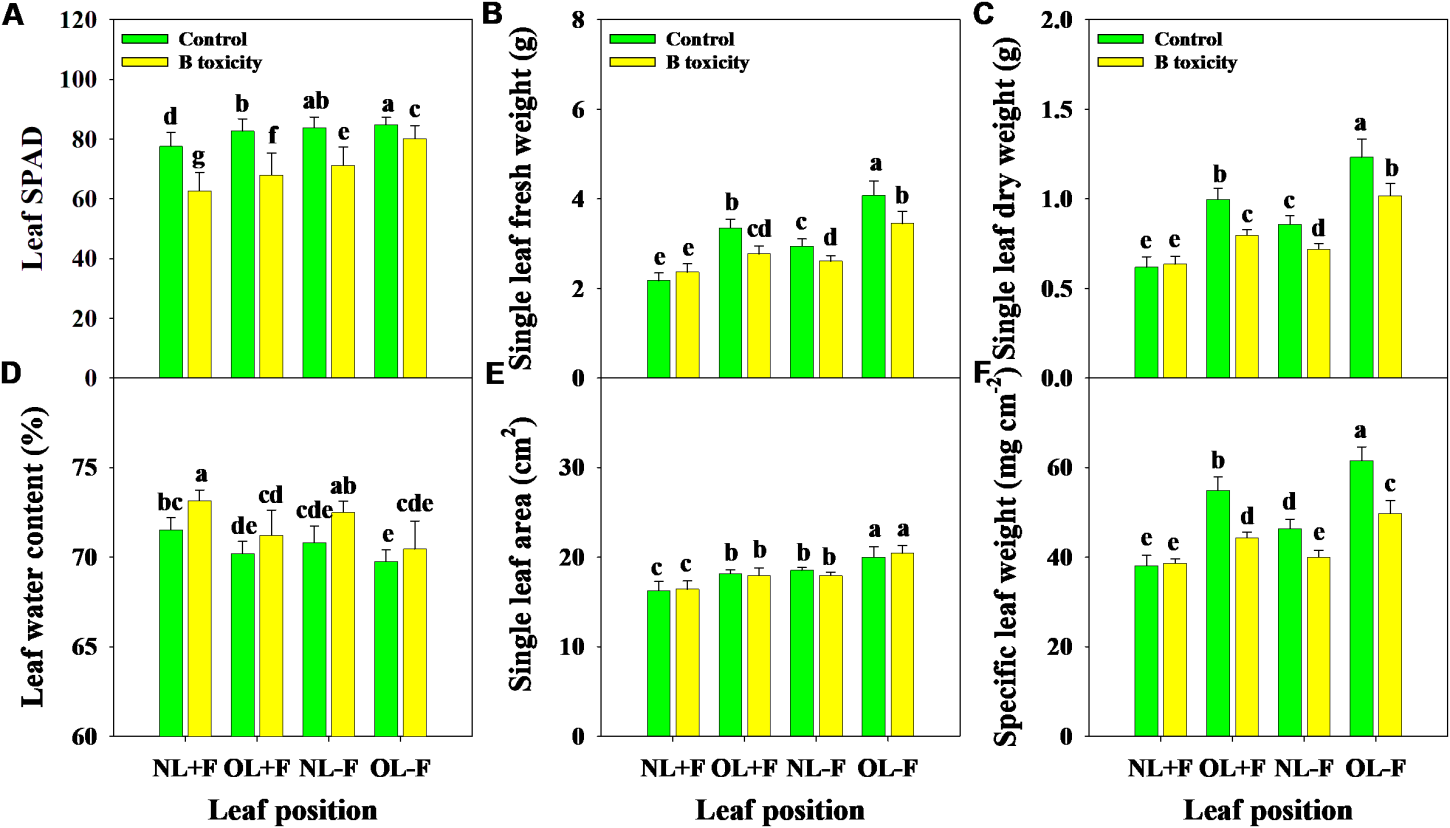
Effects of B toxicity on leaf growth variables of relative chlorophyll content [soil plant analysis development (SPAD) **A**], single leaf fresh weight **(B)**, single leaf dry weight **(C)**, leaf water content **(D)**, single leaf area **(E)**, and specific leaf weight (SLW; **F**) in different leaf positions of new leaves with fruit (NL+F), old leaves with fruit (OL+F), new leaves without fruit (NL−F), and old leaves without fruit (OL−F) in pomelo trees. The error bars indicate standard deviation, statistical analysis was carried out by ANOVA plus least significant difference (LSD) test, and statistical significance (*p*< 0.05) is indicated with different lowercase letters (a–g) in the different leaf positions.

Excess B fertilization directly led to soil total B and water-soluble B concentrations dramatically increasing (**Figure 1**), resulting in B toxicity in pomelo trees (**Figure 2**). Also, the chlorosis symptoms of B toxicity first appeared in the leaf tip and extended to the middle and bottom parts in different leaf positions, subsequently affecting leaf growth variables (**Figure 2**). B toxicity reduced leaf SPAD value but increased leaf water content across leaf positions (**Figures 3A, D**). Except for NL+F, B toxicity significantly inhibited the single leaf fresh weight and dry weight (**Figures 3B, C**), with no differences in single leaf area (**Figure 3E**), resulting in decreased specific leaf weight (**Figure 3F**). However, the leaf SPAD, fresh weight, dry weight, leaf area, and specific leaf weight in old leaves were consistently higher than those of new leaves, in contrast to those in leaf water content.

Compared with those in the control, the fruit load, single fruit weight, and fruit yield per tree in B toxicity treatment were decreased by 30.6%, 21.4%, and 45.3%, respectively (**Table 1**). Under B toxicity conditions, the fruit’s vertical and horizontal diameters were significantly decreased but did not affect the fruit shape index and edible rate. Also, fruit water content and dry weight of both pulp and peel parts were reduced in B toxicity, but had no influence on its proportions.

**Table 1:** Effects of B toxicity on fruit growth characteristics in pomelo trees.

Fruit growth characteristics	Control	B toxicity
Fruit load (# plant^−1^)	42.50 ± 2.07 a	29.50 ± 3.21 b
Fruit weight (kg fruit^−1^)	1.31 ± 0.13 a	1.03 ± 0.10 b
Fruit yield (kg plant^−1^)	55.69 ± 6.15 a	30.49 ± 5.56 b
Vertical diameter (mm)	169.92 ± 11.53 a	151.05 ± 10.86 b
Horizontal diameter (mm)	146.52 ± 5.69 a	134.35 ± 8.23 b
Fruit shape index	1.16 ± 0.08 a	1.13 ± 0.03 a
Edible rate (%)	70.54 ± 3.07 a	68.47 ± 4.09 a
Pulp water content (%)	88.54 ± 0.26 a	87.99 ± 0.43 b
Peel water content (%)	82.51 ± 0.57 a	81.66 ± 0.72 b
Pulp dry weight (g fruit^−1^)	106.78 ± 12.67 a	83.92 ± 9.40 b
Peel dry weight (g fruit^−1^)	67.71 ± 8.23 a	58.89 ± 8.10 b
Pulp proportion (%)	61.14 ± 3.38 a	58.79 ± 4.16 a
Peel proportion (%)	38.86 ± 3.38 a	41.21 ± 4.16 a

Values represent mean ± standard deviation, statistical analysis was carried out by ANOVA plus least significant difference (LSD) test, and statistical significance (p< 0.05) is indicated with different lowercase letters.

### 3.2 B toxicity disturbs mineral concentrations in leaf and fruit organs

**Figure 4:**
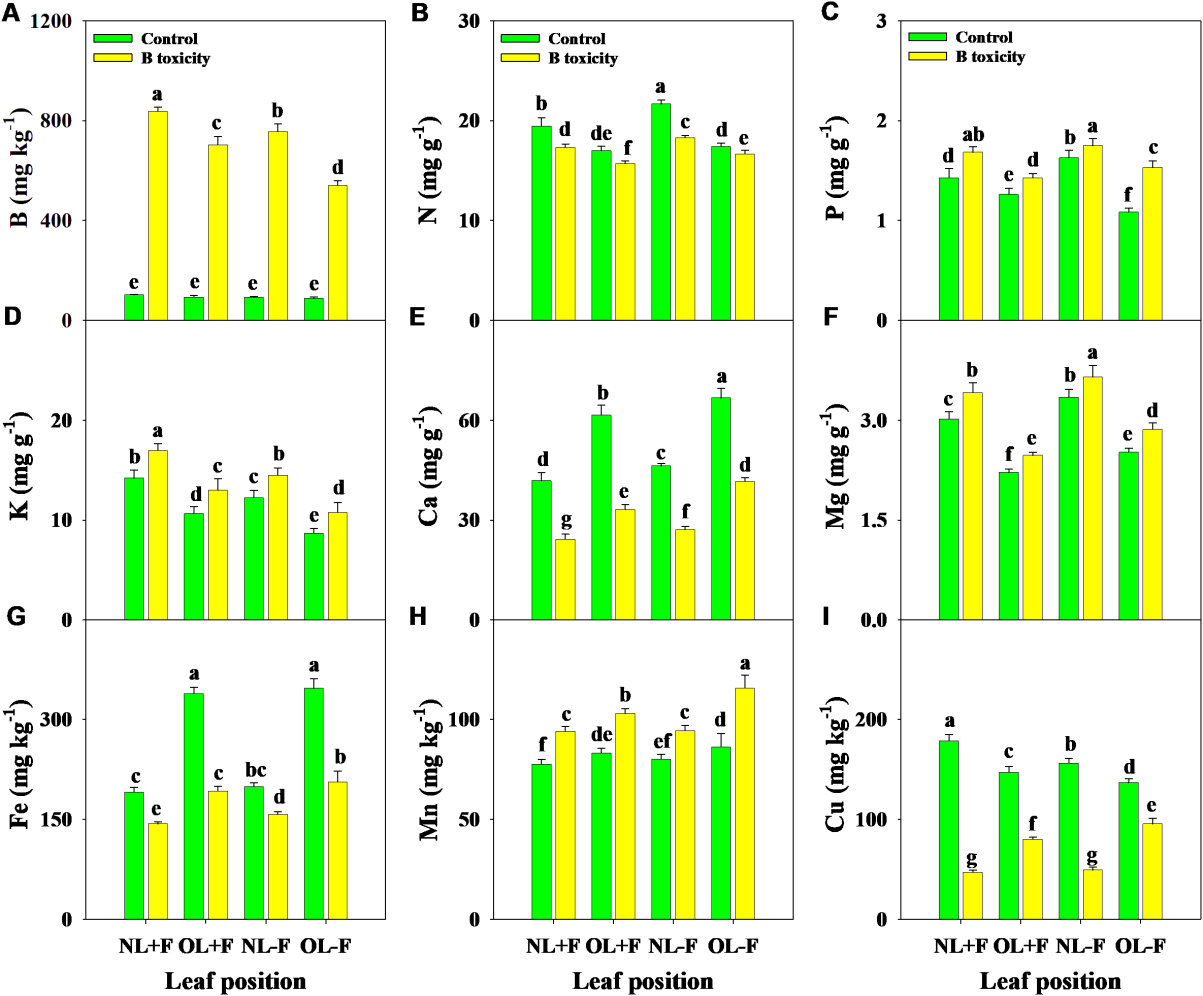
Effects of B toxicity on the concentrations of B **(A)**, N **(B)**, P **(C)**, K **(D)**, Ca **(E)**, Mg **(F)**, Fe **(G)**, Mn **(H)**, and Cu **(I)** in different leaf positions of new leaves with fruit (NL+F), old leaves with fruit (OL+F), new leaves without fruit (NL−F), and old leaves without fruit (OL−F) in pomelo trees. The error bars indicate standard deviation, statistical analysis was carried out by ANOVA plus least significant difference (LSD) test, and statistical significance (*p*< 0.05) is indicated with different lowercase letters (a–g) in the different leaf positions.

Under B toxicity treatment, the concentrations of B, P, K, Mg, and Mn in different leaf organs were increased, while N, Ca, Fe, and Cu concentrations were decreased (**Figure 4**). Regardless of the treatment, B, N, P, K, and Mg concentrations in new leaves were higher than in old leaves, in contrast with Ca, Fe, and Mn concentrations. Also, B and K concentrations were higher in leaves with fruit than in those without fruit, in contrast to N, Ca, Mg, Fe, and Mn concentrations. However, Cu concentrations in leaves with fruit were higher than without fruit under the control treatment, in contrast to the B toxicity treatment.

**Figure 5:**
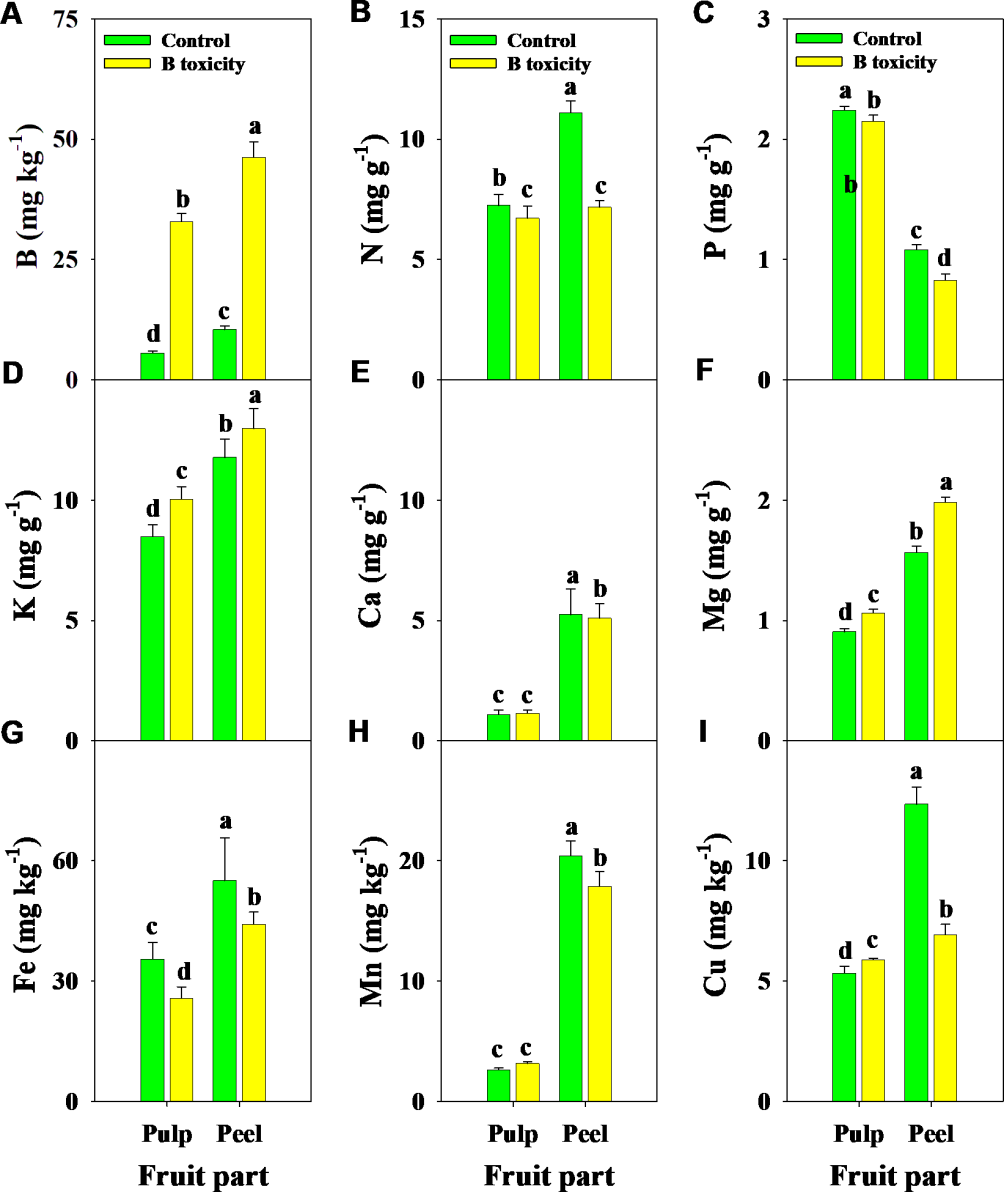
Effects of B toxicity on the concentrations of B **(A)**, N **(B)**, P **(C)**, K **(D)**, Ca **(E)**, Mg **(F)**, Fe **(G)**, Mn **(H)**, and Cu **(I)** in different fruit parts of pulp and peel in pomelo trees. The error bars indicate standard deviation, statistical analysis was carried out by ANOVA plus least significant difference (LSD) test, and statistical significance (*p*< 0.05) is indicated with different lowercase letters (a–d) in the different fruit parts.

Similarly, B, K, and Mg concentrations in different fruit parts were increased by B toxicity, but N, P, and Fe concentrations decreased (**Figure 5**). B toxicity reduced the concentrations of Ca and Mn in the peel but did not affect the pulp. Regardless of the treatment, B, N, K, Ca, Mg, Fe, and Cu concentrations were higher in the peel than in the pulp, in contrast to P. However, Cu concentrations in different fruit parts displayed an opposite trend under B toxicity, which increased in the pulp but decreased in the peel.

### 3.3 B toxicity changes leaf NSC content

**Figure 6:**
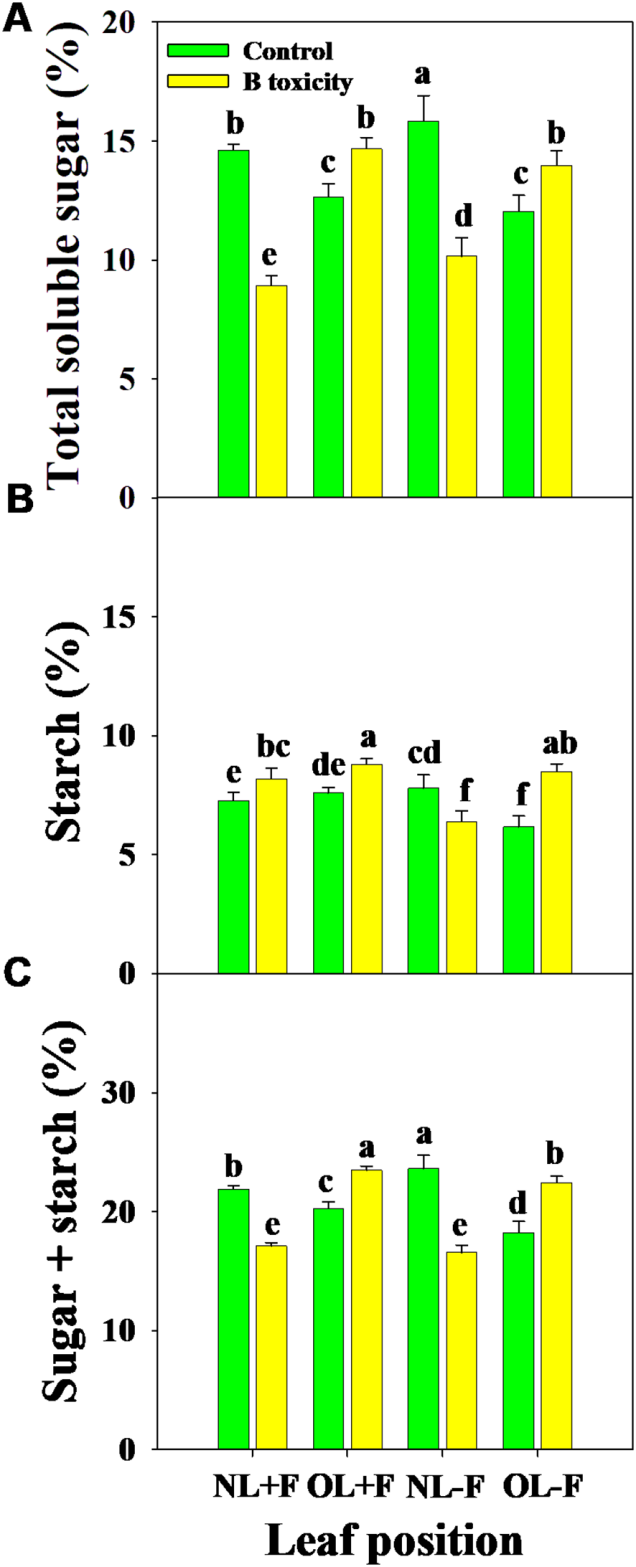
Effects of B toxicity on the content of total soluble sugar **(A)**, starch **(B)**, and non-structural carbohydrate (NSC; total soluble sugar + starch; **C**) in different leaf positions of new leaves with fruit (NL+F), old leaves with fruit (OL+F), new leaves without fruit (NL−F), and old leaves without fruit (OL−F) in pomelo trees. The error bars indicate standard deviation, statistical analysis was carried out by ANOVA plus least significant difference (LSD) test, and statistical significance (*p*< 0.05) is indicated with different lowercase letters (a–f) in the different leaf positions.

Regardless of the fruits, B toxicity reduced the total soluble sugar content in new leaves but increased it in old leaves (**Figure 6A**), and starch content increased in all leaf positions (**Figure 6B**). This resulted in increased NSC content in old leaves but decreased in new leaves (**Figure 6C**). However, the content of starch was higher than that of total soluble sugar across treatments and leaf positions.

### 3.4 B toxicity reduces fruit quality parameters

**Figure 7:**
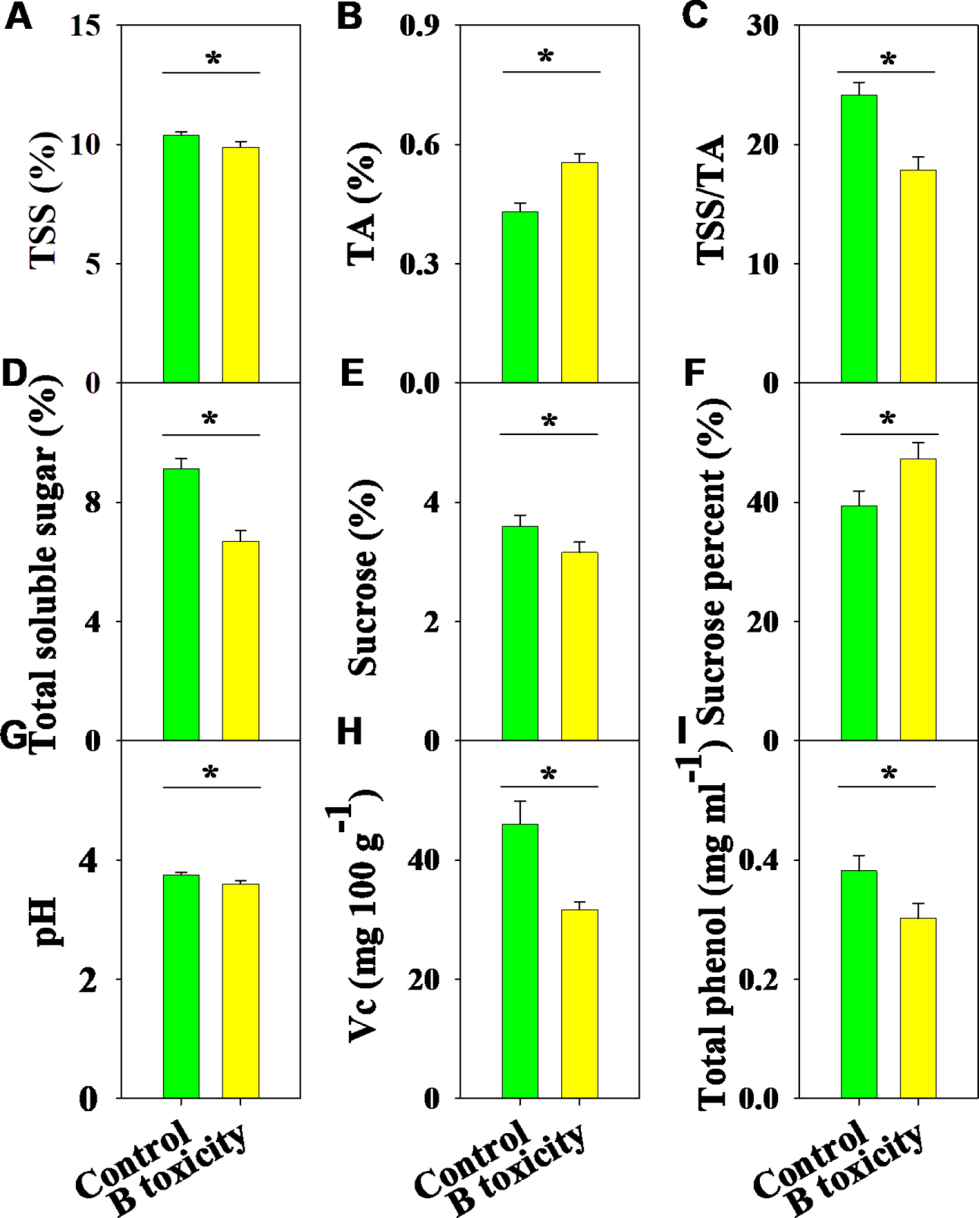
Effects of B toxicity on fruit quality characteristics of total soluble solids (TSS; **A**), titratable acidity (TA; **B**), TSS/TA **(C)**, total soluble sugar **(D)**, sucrose **(E)**, sucrose percent (sucrose/total soluble sugar × 100; **F**), pH **(G)**, vitamin C (Vc; **H**), and total phenol **(I)** in pomelo trees. The error bars indicate standard deviation, statistical analysis was carried out by ANOVA plus t-test, and statistical significance (*p< 0.05) is indicated in the different treatments.

There were significant differences in fruit quality parameters between B treatments (**Figure 7**). Under B toxicity conditions, TSS content decreased while TA content increased, resulting in a reduced TSS/TA ratio (**Figures 7A-C**). Meanwhile, the contents of total soluble sugar and sucrose decreased, but the percentage of sucrose improved (**Figures 7D-F**). Also, fruit quality parameters, including pH, Vc, and total phenol, were significantly reduced by B toxicity (**Figures 7G-I**).

**Figure 8:**
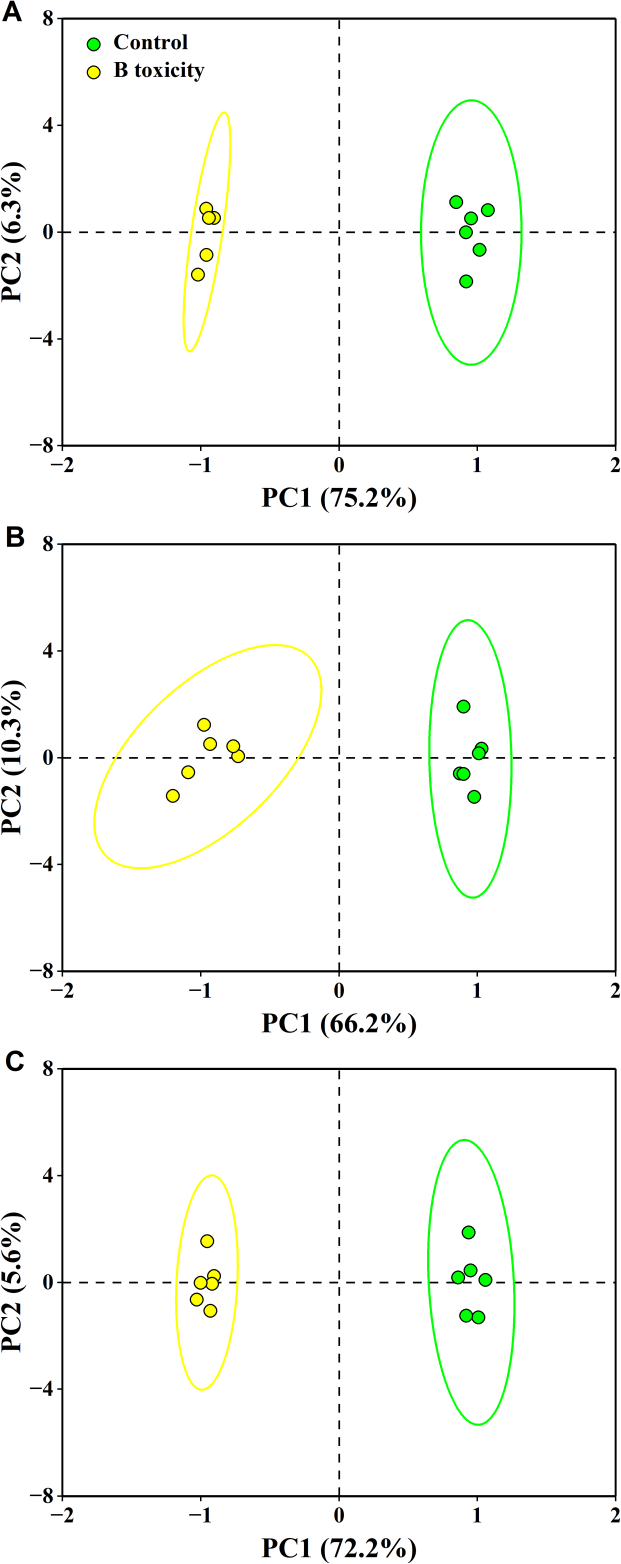
Principal component analysis (PCA) of measured parameters for leaf **(A)**, fruit **(B)**, and leaf + fruit **(C)** of both control and B toxicity in pomelo trees.

The PCA results showed that the first two components accounted for 81.5% (75.2% for PC1 and 6.3% for PC2), 76.5% (66.2% for PC1 and 10.3% for PC2), and 77.8% (72.2% for PC1 and 5.6% for PC2) of the total variation in leaf, fruit, and leaf + fruit, respectively (**Figure 8**). These measured parameters were significantly separated in different organs between treatments.

### 3.5 B toxicity affects TFs and BCFs of B in pomelo trees

**Figure 9:**
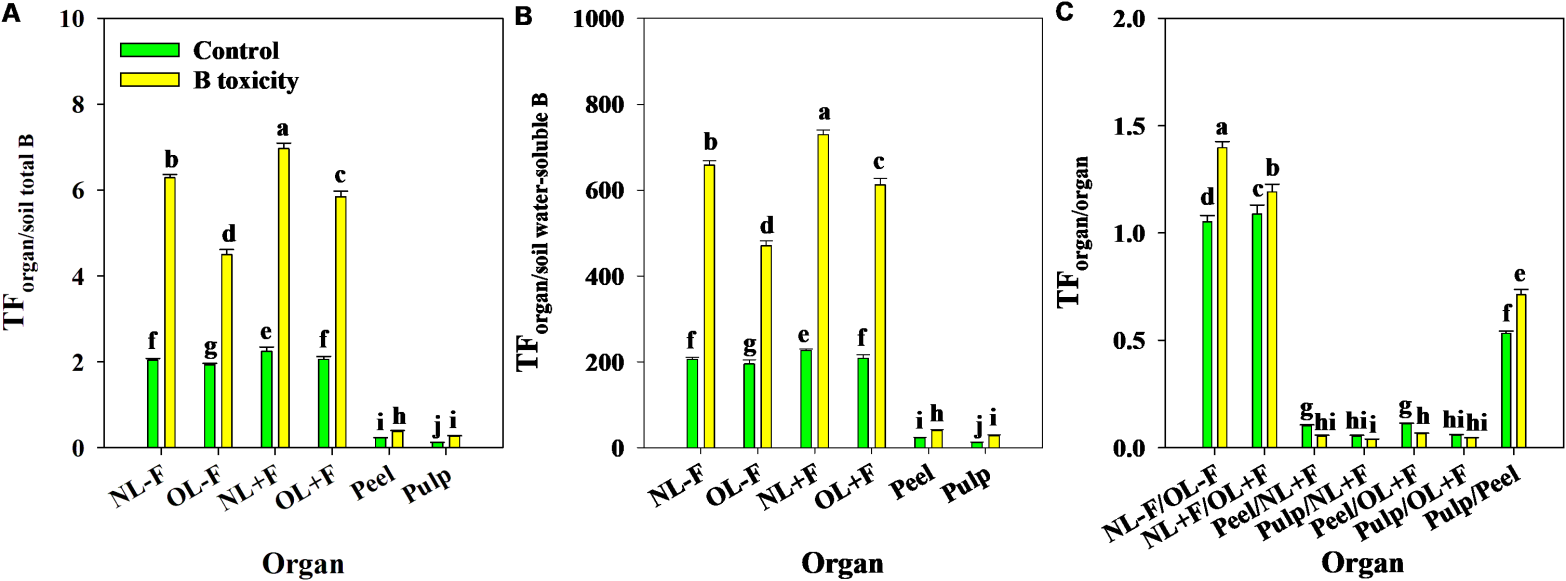
The transfer factors (TFs) of B from soil total B to organ **(A)**, from soil water-soluble B to organ **(B)**, and from organ to organ **(C)** in pomelo trees between control and B toxicity. The error bars indicate standard deviation, statistical analysis was carried out by ANOVA plus least significant difference (LSD) test, and statistical significance (*p*< 0.05) is indicated with different lowercase letters (a–j) in the different organs.

**Figure 10:**
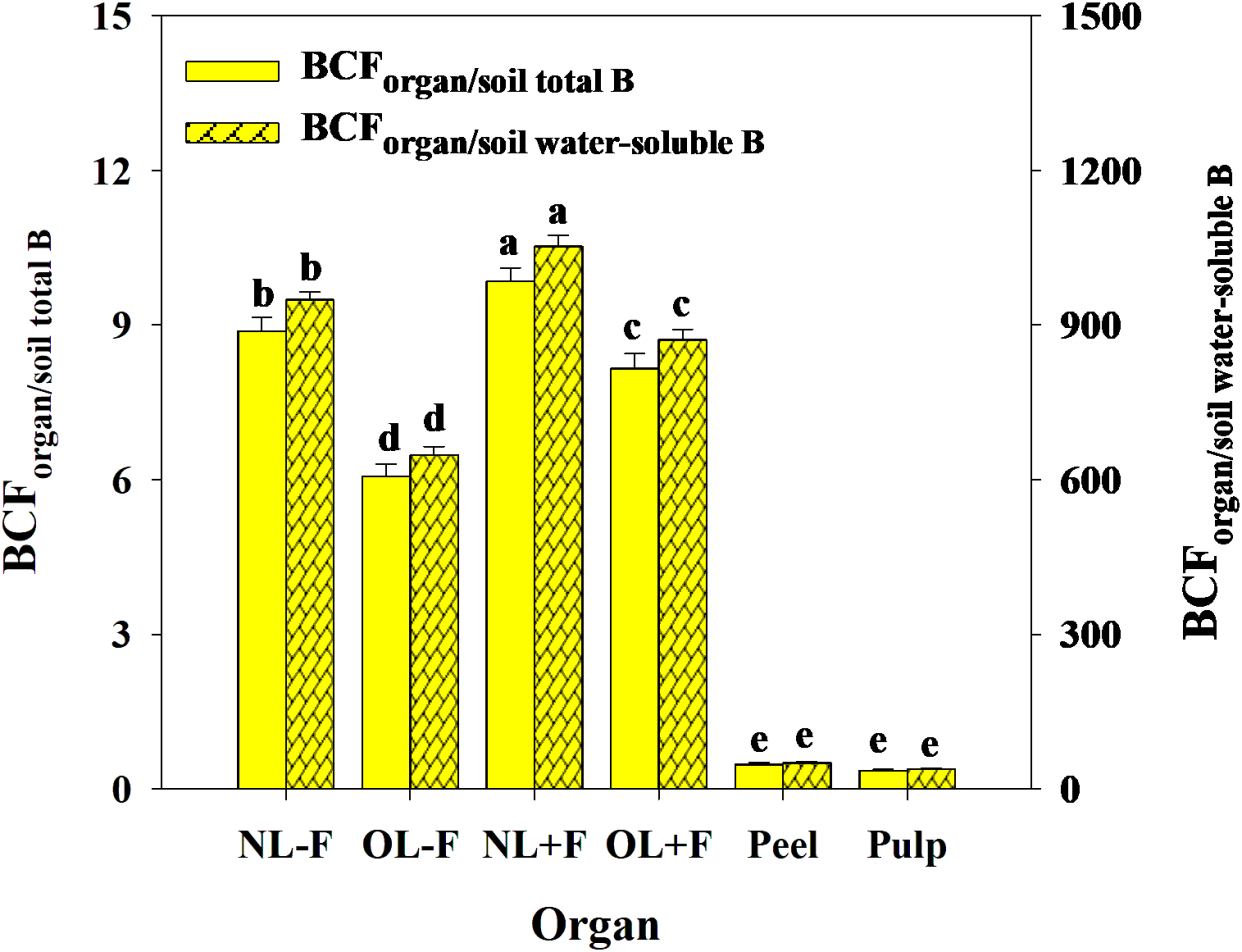
The bio-concentration factors (BCFs) of B from soil to organ in pomelo trees under B toxicity conditions. The error bars indicate standard deviation, statistical analysis was carried out by ANOVA plus least significant difference (LSD) test, and statistical significance (*p*< 0.05) is indicated with different lowercase letters (a–e) in the different organs.

B toxicity significantly increased TFs of B in pomelo plants across soil B availability and organs (**Figures 9A, B**), in which the B TFs of leaf organs (including NL−F, OL−F, NL+F, and OL+F) were higher than fruit organs (including peel and pulp) between B treatments. The B TFs of leaves with fruit (including NL+F and OL+F) were higher than those of leaves without fruit (including NL−F and OL−F), and the B TF of peel was higher than that of pulp. Also, the TFs of B between organs showed an order, as follows: from old leaves to new leaves (including TF_NL−F/OL−F_ and TF_NL+F/OL+F_) > from peel to pulp (as TF_pulp/peel_) > from leaf to peel (including TF_peel/NL+F_ and TF_peel/OL+F_) > from leaf to pulp (including TF_pulp/NL+F_ and TF_pulp/OL+F_) (**Figure 9C**). Under B toxicity conditions, the BCFs of B in different organs showed a trend of BCF_NL+F_ > BCF_NL−F_ > BCF_OL+F_ > BCF_OL−F_ > BCF_peel_ and BCF_pulp_ regardless of soil total B and soil water-soluble B, and there is no difference between BCF_peel_ and BCF_pulp_ (**Figure 10**).

**Figure 11:**
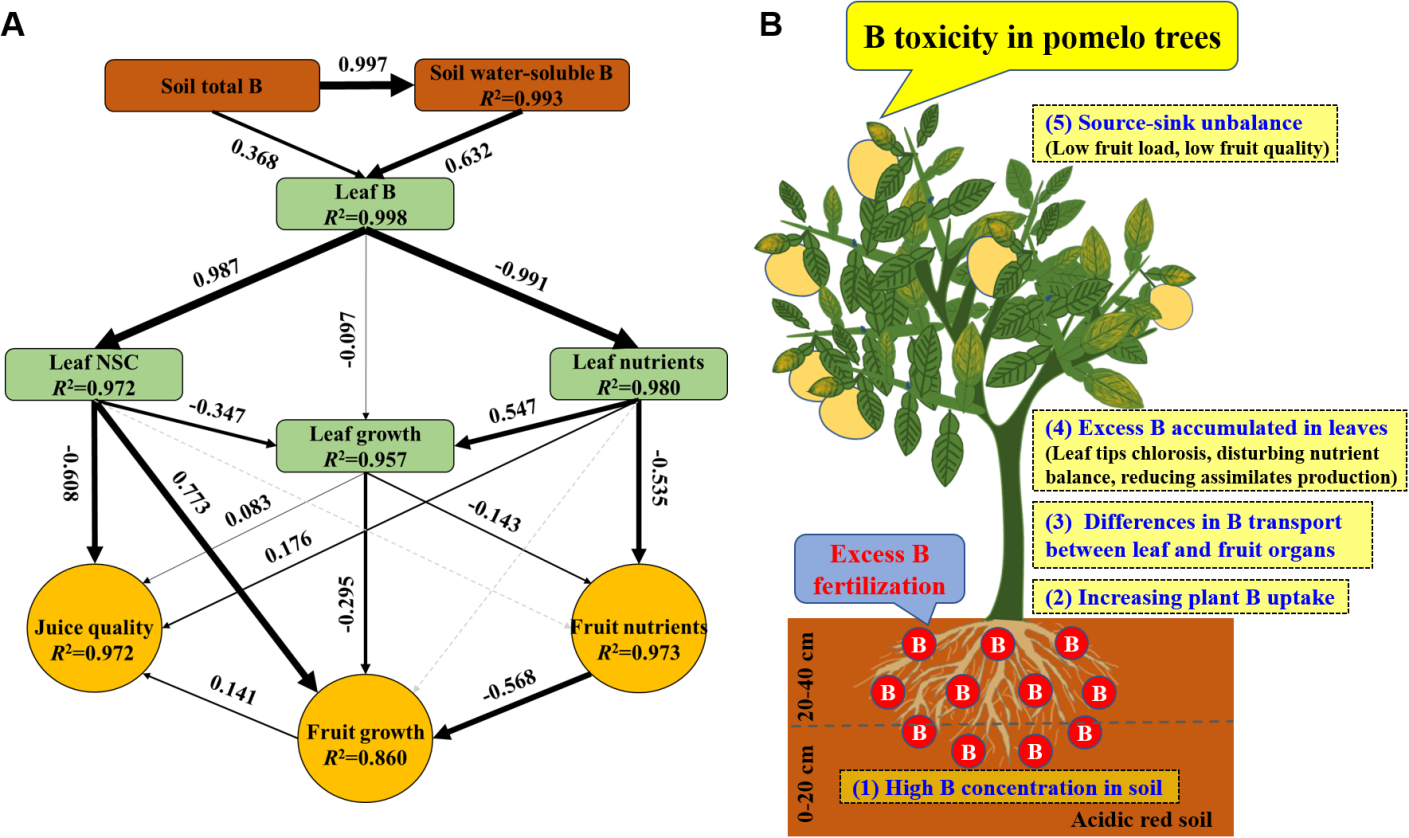
The structural equation model **(A)** and a conceptual diagram **(B)** showing the effects of excess B fertilization on plant growth, fruit yield, and quality in pomelo trees. Black arrows represent significant positive or negative pathways between variables. Gray dashed arrows represent insignificant associations between variables. Bold numbers beside arrows indicate the standard path coefficients. Arrow width is proportional to the strength of the relationship. *R* ^2^ represents the proportion of variance explained for each dependent variable in the model.

The SEM revealed that excess B fertilization reduced pomelo yield and quality through soil and plant variables (**Figure 11A**). Excess B fertilization induced changes in soil water-soluble B at 99.3% of the total variance and affected leaf B at 99.8%. Leaf B directly alters leaf NSC and nutrients at 97.2% and 98.0% of the total variance, respectively, resulting in effects on leaf growth of 95.7%. Leaf nutrients and growth explained 97.3% of the total variance of fruit nutrients. Leaf B’s direct and indirect effects on the changes in leaf NSC, growth, and fruit nutrients explained 86.0% of the total variance in fruit growth, and B-induced toxicity alters leaf characteristics (including NSC, growth, and nutrients) and fruit growth at 97.2% of the variance in juice quality. Also, a conceptual diagram of excess B fertilization-induced B toxicity with lower fruit yield and quality in pomelo trees is shown (**Figure 11B**).

## 4 Discussion

### 4.1 B toxicity reduces fruit yield and quality in pomelo trees

Most previous studies focused on the growth inhibition of citrus seedlings in response to B toxicity and have explained its physiological and molecular mechanisms, including photosynthetic, cell ultrastructure, gene expression, protein, microRNA, and transcription profiles (Huang et al., 2014, 2019; Guo et al., 2014; Sang et al., 2015, 2017; Wu et al., 2018, 2019). However, little information exists on the effects of fruit yield and quality on response in citrus trees of B toxicity under field conditions. Our results showed that B toxicity significantly decreased pomelo yield and its components, including fruit load and single fruit weight, which implies that B toxicity may reduce the fruit setting rate and damage the production and transport of assimilates for lower sink demand (Huang et al., 2014; Yang et al., 2022; Li et al., 2023). It is generally accepted that B toxicity induced leaf chlorosis, which further decreased leaf photosynthetic performance (Chen et al., 2022; Yang et al., 2022). Additionally, the leaf net photosynthetic rate (P_n_) decreased with increasing fruit sink demand in citrus plants (Zhang et al., 2013). Martínez-Alcántara et al. (2015) reported a negative correlation between fruit carbon utilization and shoot vegetable growth in citrus plants, mainly caused by photoassimilate resource limitation. As a result, the intensity of NSC supply from leaf photosynthetic production determines fruit formation in quantity and quality (Zhang et al., 2013). In the present study, the content of total soluble sugar and NSC was increased in old leaves and decreased in new leaves by B toxicity, which implied that B toxicity limits the production capacity of photosynthate in new leaves and reduces the transport capacity in old leaves whether fruiting or not. However, the starch content was always increased across leaf positions, indicating that B toxicity inhibits the transport of assimilates in leaves and accumulates in the form of starch (Huang et al., 2014). The corresponding results are also reflected in the leaf growth characteristics between B treatments, in which the old leaves had higher biomass, leaf area, and specific leaf weight than the new leaves; meanwhile, these were higher without fruit compared with fruit, and all were reduced under B toxicity. These results indicated that the fruit-bearing capacity is directly related to leaf function, and healthy leaves are the basis for ensuring sufficient assimilation supply and coordinating the relationship between source and sink for high yield and high quality in citrus production (Landi et al., 2019; Brdar-Jokanović, 2020; Yang et al., 2022).

Interestingly, the proportion of different fruit parts did not change between B treatments, indicating that the decline of peel and pulp was synchronous under B toxicity conditions, resulting in no differences in the edible rate and fruit shape index. However, the reduction in pulp dry weight (21.4%) was higher than that of peel (13.0%), which mainly reduced water content. Also, the effects of B toxicity on leaf water content were observed, which was increased in all leaf positions. The relationship between B toxicity and water uptake in plants was reported by Schnurbusch et al. (2010), who found that B toxicity tolerance was related to the gene expression of the aquaporin in the roots. Gimeno et al. (2012) reported that B toxicity decreased leaf water potential by reducing the osmotic potential in four lemon plants, which mainly increased organic solute compounds in the leaf, such as soluble sugars. Furthermore, B toxicity reduced assimilate production by inhibiting gas exchange parameters, including P_n_, stomatal conductance (g_s_), and transpiration rate (T_r_) (Papadakis et al., 2004a; Huang et al., 2014; Simón-Grao et al., 2018) but did not affect the intercellular CO_2_ concentration (C_i_) and water use efficiency (Gimeno et al., 2012; Guo et al., 2014; Sang et al., 2015). These results indicated that non-stomatal factors contributed to photosynthesis depletion, further implying that B toxicity increased leaf water content may be inhibiting g_s_ and T_r_ for reducing water loss in leaf and that decreased fruit water content was possibly caused by affecting the water transport in plants. It suggests that B toxicity may regulate water uptake, transport, and circulation in various organs of plants, including pomelo trees (Gimeno et al., 2012; Wimmer and Eichert, 2013).

B toxicity significantly decreased pomelo fruit quality, which decreased TSS/TA by increasing TA and reducing TSS, while decreased total soluble sugar and pH reflected alterations in TSS and TA. Also, B toxicity reduced pulp sucrose content and increased sucrose percent, implying that B stress regulates fruit sugar metabolism (Landi et al., 2019). However, the pulp Vc and total phenol contents were decreased consistently. Combined with the results of water and NSC contents above, B toxicity affected the material transport in the xylem and phloem, further enhancing its negative effect on fruit quality (Landi et al., 2019). Furthermore, roots are the most sensitive organs surrounding the toxic rhizosphere and rapidly respond to unfavorable niches and abiotic stresses, including excess B. Although our results did not focus on the responses of the root, previous studies reported that B toxicity induced cell wall structure disorder, anatomical damages, cell elongation reduction, and abnormal apical enlargement in roots, eventually affecting its growth and function (Huang et al., 2014, 2019; Shah et al., 2017; Li et al., 2023).

### 4.2 B toxicity alters the nutrient balance in pomelo trees

Generally, B concentration in citrus plants showed a trending order of leaf > root > stem, in which the basal leaves > middle leaves > upper leaves, but there was a difference between the stem of rootstock and scion (Papadakis et al., 2004a; Sheng et al., 2009). Simón-Grao et al. (2018) reported that excess B supply directly led to a dramatic increase in B concentration in organs, further affecting physiological and biochemical processes in citrus plants (Papadakis et al., 2004a, b; Yang et al., 2022). Boaretto et al. (2011) found that citrus leaf was the dominant organ of B accumulation compared to fruits, and the B concentration in young leaves was higher than that in old leaves, resulting in the symptom of B toxicity first developing in old leaves, especially in leaf tips (Wu et al., 2018, 2019; Landi et al., 2019). Similar results were observed in the present study, which also showed that B concentration in the leaves with fruit was higher than without fruit, and in peel, it was higher than that in pulp. Liu et al. (2011) reported that B accumulates mainly in leaf cell walls and may intrude into the cytoplasm, thus disturbing cytoplasmic metabolism and causing B toxicity. However, the capacity of B uptake in citrus plants was regulated by rootstock (Papadakis et al., 2004a, b; Sheng et al., 2009; Simón-Grao et al., 2018). These results can explain that B concentration in differences between the new and old leaves may be related to the leaf ages and that higher B concentrations in the fruiting leaf and peel are caused by higher physiological activity as the growth center for fruit formation (Boaretto et al., 2011). Liu G. D. et al. (2012) reported that newly absorbed B in citrus plants was preferentially transported to new leaves through a ^10^B isotope experiment. The most direct response of pomelo plants to excessive B supply was luxury B uptake, reflected in extreme B concentrations in leaf and fruit organs, resulting in leaf chlorosis and decreased SPAD value in all leaf positions. Similar results were also observed by Wu et al. (2018, 2019), who reported that B toxicity preferentially manifested in the leaf tip, but the leaf center remained green in lower leaves. Landi et al. (2019) pointed out that B is mainly translocated to mature leaves through non-living xylem cells driven by transpiration, implying that the factors affecting transpiration also regulate B absorption and transport in citrus plants.

Alteration of nutrient profiles is an inevitable process of plants responding to environmental stresses, especially nutrient stress. Riaz et al. (2018a) reported that Ca concentrations in the root and stem of citrus seedlings were decreased with increasing B supply. Except for increased K concentration, excess B did not alter the concentrations of N, P, Ca, and Mg in the leaf of lemon trees grafted on four rootstocks, and N and Ca in roots were decreased but did not affect P, K, and Mg (Gimeno et al., 2012). Guo et al. (2014) reported that B toxicity decreased leaf P concentration in high B-sensitive citrus, implying that plant nutrient status is related to B toxicity tolerance. Similar results were found by Kaya et al. (2009), who reported that B toxicity decreased the concentrations of Ca, P, and K in tomato leaves. These results indicated an antagonism between B and P, K, and Ca in plants (Li et al., 2023). However, our results showed that the concentrations of P, K, Mg, and Mn were increased and those of N, Ca, Fe, and Cu were decreased in all leaf positions under B toxicity, and the concentrations of N, P, and Fe were decreased and those of K and Mg were increased in pulp and peel parts. These results further indicated that the homeostasis of mineral elements in plants was changed by B toxicity, which could have contributed to the lower tolerance for excess B and finally reduced plant growth and fruit quality.

### 4.3 Characteristics of B transport from soil to fruit in pomelo orchards

The characteristics of B transport in soil–plant systems are closely related to the B absorption and distribution features, which can reveal the adaptive response of plants to B deficiency or excess stress (Liu G. D. et al., 2011, 2012; Landi et al., 2019; Hua et al., 2021). The TF and BCF are two critical indicators used to analyze the ability of plants to absorb and transport a given element from soil and organs (Zhang et al., 2018). In the present study, the TFs of B in leaf organs (such as TF_NL−F_, TF_OL−F_, TF_NL+F_, and TF_OL+F_) were always higher than those in fruit organs (such as TF_peel_ and TF_pulp_), and the process was strengthened by B toxicity regardless of soil B availability. Also, the TFs of B between organs showed three classifications: TF_NL−F/OL−F_ and TF_NL+F/OL+F_ were the highest, followed by TF_pulp/peel_, and then TF_peel/NL+F_ and TF_peel/OL+F_, TF_pulp/NL+F_, and TF_peel/OL+F_. These results indicated that less B was accumulated in the fruit as compared to that in other organs in pomelo plants between B treatments, in which the capacity of B transport from old leaves to new leaves was higher than that from peel to pulp and had a lower B transport from leaf to fruit (including peel and pulp). It is consistent with results reported by Boaretto et al. (2008, 2011), who estimated that 30%–35% of total B in the new leaves remobilized from other reserve organs of citrus plants, and a lower B concentration in fruits means lower transport capacity than in leaves. Similarly, the BCFs of B in the new leaf were higher than those in the old leaf under B toxicity conditions, and the leaf with fruit was higher than the leaf without fruit across soil B availability; however, the BCFs of B in leaf organs were always higher than those in fruit organs. These results indicated that the leaf was the main organ of B accumulation and showed a fence effect in the transport of B from leaf to fruit under B toxicity conditions. It further implied that as a species with restricted phloem mobility, the loading capacity of B from source (as leaf) to sink (as fruit) was limited in pomelo plants. Regardless of the soil B availability and B treatments, the TFs and BCFs of B in the leaves with fruit were consistently higher than those in leaves without fruit, indicating that the fruit load regulates leaf B uptake, and the leaves bearing fruit are the growth center of pomelo tree, which have stronger physiological and metabolic functions (Zhang et al., 2013; Martínez-Alcántara et al., 2015). There are higher TF and BCF of B in peel compared with those in pulp, which can be related to peel as a source organ with a stronger gas exchange function during citrus fruit development, whereas pulp is a pure sink organ (Hiratsuka et al., 2015).

Furthermore, the TFs of B were >1 from soils to leaves and<1 from soils to fruits, indicating a difference in B transport between leaf and fruit organs, and the process was enhanced by excess B application. Also, the TFs of B from organ to organ (TF_organ/organ_) were much less than those of B from soil to organ (TF_organ/soil_), which means that the absorption capacity of pomelo organs to soil B was higher than the reutilization capacity of B between organs. However, the TF_pulp/peel_ of B was consistently higher than the TFs of B from leaves to fruits. Moreover, the BCFs of B were >1 from soils to leaves and<1 from soils to fruits under B toxicity conditions, indicating a higher B concentration in pomelo leaves and a lower B concentration in pomelo fruits than in soils. Also, the TFs and BCFs based on soil total B and water-soluble B showed a trend change, which was consistent with the findings of Wang et al. (2006), who reported that there was a significant relationship between the TFs and the corresponding soil metal concentrations, including total and diethylene triamine pentaacetic acid (DTPA)-extractable metal contents. Generally, the study demonstrated that various pomelo organs show a different absorptive ability of B, and pomelo leaves have a greater ability to absorb B than pomelo fruits. Differences in TFs and BCFs between organ/soil and organ/organ further indicate that the B concentration varied widely in different parts of the pomelo plants. Previous studies reported the characteristics of scion–rootstock interaction, fertilizer, and water management in the soil environment and the compartmentalization and translocation in the vascular system, which may be related to B variability in different organs of plants, including pomelo (Landi et al., 2019; Brdar-Jokanović, 2020; Du et al., 2021; Hua et al., 2021).

### 4.4 Optimizing B management for sustainable citrus production in acidic B deficiency soil conditions

Understanding how B management impacts fruit yield can be helpful for sustainable citrus production, in which high-quality fruit is the basis of maintaining the demand for consumption growth and diet improvement for human health (Wang et al., 2019; Du et al., 2021; Cheng et al., 2022). It is widely accepted that soil B availability and plant B requirement were critical indexes for optimizing B management in citrus production (García-Sánchez et al., 2020). Guo et al. (2022) reported that soil available B coexisted with excessive and deficiency in pomelo orchard in this study area. In general, the value of available B in soils ranges from 0.5 to 5 mg kg^-1^, with B deficiency occurring below 0.5 mg kg^-1^ and toxicity occurring higher than 5.0 mg kg^-1^ (Shah et al., 2017; Brdar-Jokanović, 2020; Thakur et al., 2023). Krug et al. (2023) proposed that the critical level and sufficiency ranges of B concentration in citrus leaves for fruit production were 117 mg kg^-1^ and 85–149 mg kg^-1^, respectively. Reasonable application of B-contained fertilizer, including soil and foliar spray, can significantly improve citrus yield and quality in B-deficient citrus orchards (Mattos-Jr et al., 2017). Boaretto et al. (2011) reported that the boric acid applied in soil (as 1.0–2.0 kg ha^−1^) was more efficient than in foliar (as 0.5–1.0 kg ha^−1^) in citrus orchards. Also, optimizing B application can mitigate citrus root injuries in response to environmental stress, such as soil acidification (Yan et al., 2019), aluminum stress (Riaz et al., 2018b; Yan L. et al., 2018, 2024), and copper toxicity (Chen et al., 2022, 2023, 2024). Therefore, adequate B nutrition is indispensable for better citrus trees and higher fruit yield, and quantitative monitoring of B nutritional status in soils and trees is crucial for targeted B fertilization to avoid toxicity.

Corresponding to correct B deficiency, B toxicity often occurs in citrus production, inevitably affecting plant growth and yield formation. Also, it was difficult to control when plants were exposed to an excess B environment. Papadakis et al. (2004a, b) found that B toxicity tolerance was related to citrus varieties, which have higher tolerance varieties mainly through reducing B absorption and maintaining more B in stems and roots, thus decreasing B transport to leaves. A similar result has been reported that B tolerance in citrus varieties is related to the balance of B forms among free, semi-bound, and bound B (Liu et al., 2011). However, B absorption, transport, and tolerance of citrus plants were affected by varieties, rootstocks, and environment (Gimeno et al., 2012; Huang et al., 2014; Simón-Grao et al., 2018; García-Sánchez et al., 2020; Du et al., 2021). In connection with the above-mentioned nutritional responses to B toxicity in different plants, it also provides a strategy for citrus trees to regulate B toxicity through antagonistic characteristics among mineral elements, such as supplementary N-, P-, and Ca-contained fertilizers (Kaya et al., 2009; Li et al., 2023). Hua et al. (2021) summarized three mechanisms to alleviate B toxicity in plants from the perspective of soil–plant systems, including decreasing tissue B concentration and cellular B activity and increasing physiological tolerance. Correspondingly, three alleviative approaches are proposed that apply nutrient elements, plant growth regulators, and plant growth-promoting microbes (Hua et al., 2021). In any case, these measures in the control of citrus B toxicity need to be further studied. Interestingly, our later investigation showed that the productivity of B toxicity pomelo trees almost completely recovered after 2 years (data not shown). A similar result was observed by Boaretto et al. (2011), who found that the residual effect of B fertilization was drastically reduced in the next year, which may be related to the B removed by the leaf abscission, pruning branches, and fruit harvest from the citrus tree and leaching losses from the soil. Therefore, developing integrated B management based on soil–plant–environment systems is the key to ensuring high-yield and high-quality citrus production.

## 5 Conclusions

Considering the effects of B toxicity on pomelo yield and fruit quality, a field experiment was conducted to investigate the differential response between control and excess B fertilization. As a pattern summarized above, excess B fertilization directly induced B toxicity in pomelo trees through dramatically increased plant B uptake and transport. B toxicity reduces leaf growth and related physiological characteristics by reducing the assimilate production in new leaves and decreasing its transport capacity in old leaves. B toxicity reduces fruit yield and quality formation by disturbing mineral homeostasis in various leaf positions and fruit parts. Moreover, the TFs and BCFs of B in leaf organs were higher than those in fruit organs and intensified by B toxicity. In conclusion, our findings reveal the absorption and mobility of B in pomelo plants and provide essential information for optimizing B management in pomelo green production.

## Data Availability

The original contributions presented in the study are included in the article/supplementary material. Further inquiries can be directed to the corresponding author.
